# Ecology of *Legionella pneumophila* biofilms: The link between transcriptional activity and the biphasic cycle

**DOI:** 10.1016/j.bioflm.2024.100196

**Published:** 2024-03-30

**Authors:** Ana Barbosa, Nuno F. Azevedo, Darla M. Goeres, Laura Cerqueira

**Affiliations:** a LEPABE – Laboratory for Process Engineering, Environment, Biotechnology and Energy, Faculty of Engineering, University of Porto, Rua Dr. Roberto Frias, 4200-465, Porto, Portugal; b ALiCE – Associate Laboratory in Chemical Engineering, Faculty of Engineering, University of Porto, Rua Dr. Roberto Frias, 4200-465, Porto, Portugal; c The Center for Biofilm Engineering, Montana State University, Bozeman, MT, USA

**Keywords:** Biofilm ecology, Spatial organization, Cell functionality, *Legionella pneumophila*, Biofilm physiology

## Abstract

There has been considerable discussion regarding the environmental life cycle of *Legionella pneumophila* and its virulence potential in natural and man-made water systems. On the other hand, the bacterium's morphogenetic mechanisms within host cells (amoeba and macrophages) have been well documented and are linked to its ability to transition from a non-virulent, replicative state to an infectious, transmissive state.

Although the morphogenetic mechanisms associated with the formation and detachment of the *L. pneumophila* biofilm have also been described, the capacity of the bacteria to multiply extracellularly is not generally accepted. However, several studies have shown genetic pathways within the biofilm that resemble intracellular mechanisms. Understanding the functionality of *L. pneumophila* cells within a biofilm is fundamental for assessing the ecology and evaluating how the biofilm architecture influences *L. pneumophila* survival and persistence in water systems. This manuscript provides an overview of the biphasic cycle of *L. pneumophila* and its implications in associated intracellular mechanisms in amoeba. It also examines the molecular pathways and gene regulation involved in *L. pneumophila* biofilm formation and dissemination. A holistic analysis of the transcriptional activities in *L. pneumophila* biofilms is provided, combining the information of intracellular mechanisms in a comprehensive outline. Furthermore, this review discusses the techniques that can be used to study the morphogenetic states of the bacteria within biofilms, at the single cell and population levels.

## 1 Introduction

*Legionella* is an intracellular Gram-negative facultative pathogen found in natural aquatic environments worldwide (e.g., lakes, rivers, creeks, hot springs, and other bodies of water), associated with freshwater protozoa [[1], [2], [3]]. However, the colonization of artificial water systems like cooling towers, spa pools and showers can create environments suitable for the growth and spread of *Legionella,* in particular *Legionella pneumophila*, leading to human infections [1,4,5].

In recent years, much has been inferred about the persistence of *Legionella* in water systems [6,7]. The opportunistic nature of this bacterium allows the adaptation to various ecosystems, making it a subject of extensive study as a pathogen [2]. *Legionella* was the etiological agent of numerous outbreaks worldwide, resulting in considerable costs related to hospitalizations and industrial losses [8,9]. Of the 72 *Legionella* species, most human infections are associated with *L. pneumophila* [[10], [11], [12]]. *L. pneumophila* was first identified as being pathogenic to humans after an outbreak of acute pneumonia at a convention of the American Legion in Philadelphia, USA in 1976, and has since been recognized as the main etiologic agent of Pontiac fever and Legionnaire's Disease (LD), two forms of respiratory illnesses [[13], [14], [15]].

**Fig. 1:**
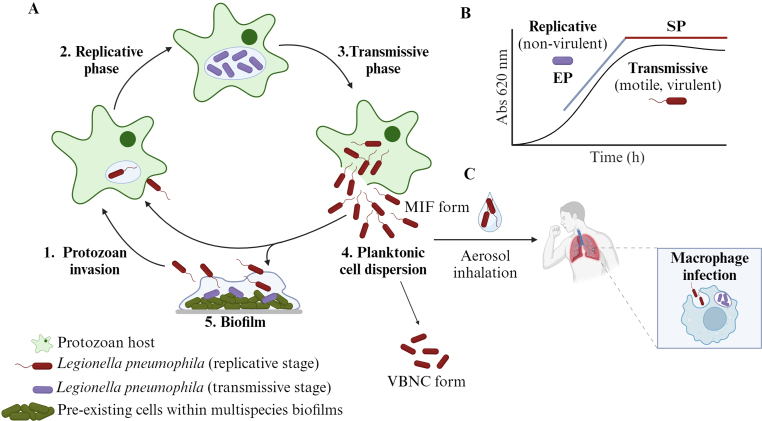
*Legionella pneumophila* within intracellular and extracellular environments. **(A)** Freshwater ecosystems. **1.** Invasion of protozoan host by a free living planktonic cell. **2.** After internalization, the bacterium evades the phagosome-lysosome fusion and starts the intracellular multiplication within the *Legionella*-containing vacuole (LCV) (replicative phase). **3.** After the nutrient's conditions become limited, the bacteria switch to a transmissive phase**. 4.** New cells can lyse the vacuolar membrane and are released in the extracellular environment. Free-living transmissive bacteria can start a new cycle **(1)**, associate with biofilms **(5)** or persist in the extracellular environment as viable but non-culturable (VBNC). **(B).** In nutrient-rich medium, *L. pneumophila* presents a biphasic life cycle, that resembles the replicative (exponential phase (EP) and transmissive intracellular forms (stationary phase (SP)). **(C)** Release of the mature infectious form (MIF) from the protozoan host can cause Legionnaire's disease through inhalation of aerosols in humans. Created with BioRender.com.

One of the largest outbreaks reported in Europe occurred in Portugal in 2020 with 88 confirmed cases of Legionnaire's Disease and 13 deaths linked to industrial cooling towers [16]. Indeed, cooling towers have been identified as one of the sources of contamination [17]. The ubiquity of *L. pneumophila* and its link to human health underpins the importance of understanding what triggers infection and motivates the bacterium's virulent behaviour. While not straightforward, the answer can likely be found by its complex ecology and resilience, as *L. pneumophila* can metabolically, physiologically and morphologically adapt as free-living cells, embedded in biofilms or inside host cells (protozoa and macrophages). This enables this pathogen to survive in nutrient-poor environments and inside hosts, as well as denote an increasing resistance to antimicrobials and disinfectants [18,19] (Fig. 1).

All these physiological transitions imply morphogenetic changes in the bacterial cell wall, shape and motility [20]. In these water niches, the molecular pathways involved in the amoeba intracellular mechanisms, within the replication and transmissibility mechanisms (see below the biphasic cycle section) have been well identified and are linked to the pathogenesis of the bacterium and its ability to invade and delve into host cells [[21], [22], [23]]. Although the regulatory pathways and genes associated with biofilm growth are being documented [24,25], little is known about the ecology of *L. pneumophila* and the cellular functionality within the biofilm matrix under different conditions. This poses the question: why do biofilm cells trigger bacterial pathogenicity? The behaviour of *L. pneumophila* in water biofilms can be affected by various physicochemical parameters such as surface, temperature, iron concentration, and the presence of biocides. Additionally, biological factors such as the composition of the biofilm population can either enhance or hinder the bacteria's persistence [24].

At the cellular scale, external factors govern the formation of *L. pneumophila* biofilm at the transcriptional level [24,26]. In fact, there is a resemblance between biofilm metabolic expression and intracellular survival mechanisms (replicative/transmissive states), and some genes/molecular pathways involved might be associated with the morphological states within a biofilm that may be linked to virulence [27]. While few studies have found evidence to suggest that the bacterium can reproduce extracellularly [[28], [29], [30]], it has been shown that *L. pneumophila* does use eukaryotic cells to reproduce and complete the cell cycle [[31], [32], [33]]. Besides that, in biofilms, the non-growing cells of *L. pneumophila*, present metabolic activity and express virulence genes [27].

As such, knowing the spatial location of cells and the phenotypic diversity at the single-cell level [34,35], is of great importance, since deconstructing how biofilm functions in a given environment may be key to understanding what strategies can be used to monitor, control and eradicate biofilms in water systems. Fortunately, the knowledge on biofilm dynamics and complexity at the single-cell level has been greatly evolving in the past years accompanying the advances in technologies at the analytical, molecular and imaging level [36]. This allowed for the study of gene expression (e.g. RT-qPCR) [37], and spatial organization (e.g. GFP staining; FISH techniques) [38] within these microbial communities structures under different environmental circumstances.

Herein, we will start explaining the concepts behind the *L. pneumophila* biphasic cycle, providing a framework for the associated intracellular mechanisms in amoeba. Afterwards, the molecular pathways and gene regulation involved in *L. pneumophila* biofilm formation and dissemination into new niches will be explored, harmonizing the acquired knowledge of these regulatory pathways with the biphasic life cycle in host cells. These insights may help to explain not only the virulence state of the bacteria detached from a biofilm, but also contribute to the debate of the possible extracellular replication of *L. pneumophila*. Finally, state-of-the-art detection technologies that may be used to further understand *L. pneumophila* ecology in water systems biofilms will be described.

## *2* *L. pneumophila* biphasic life cycle

**Fig. 2:**
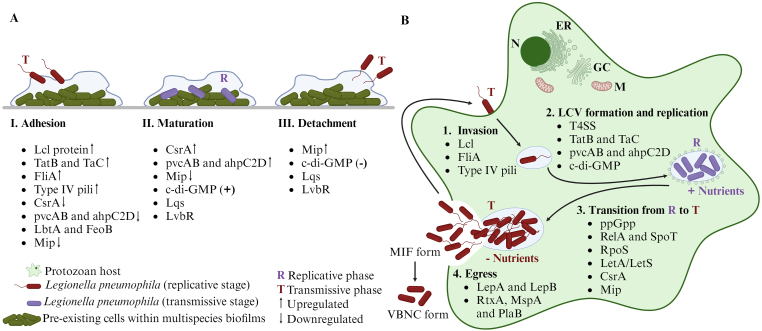
**- (A)***Legionella pneumophila* within multispecies biofilms. **I.***L. pneumophila* adheres to a pre-existing biofilm. **II.** The cells find a favourable environment which allows them to repress some virulence genes. **III**. When favourable conditions are depleted, *L. pneumophila* starts expressing virulence genes and detaches from the biofilm. **(B)** Intracellular regulation during host protozoan infection. **1**. The bacteria adhere to the host cell through phagocytosis mechanisms. **2**. The T4BSS effector proteins and other factors allow the establishment of the LCV, and the bacteria initiate the multiplication (replicative phase). **3**. When nutrients become limiting, the bacteria switch to a transmissive phase. **4.** Bacteria are released into the cytosol, as mature infectious form (MIF) or enter the viable-but-not-culturable (VBNC) form. **N**: nucleus; **GC**: Golgi complex; **ER:** endoplasmic reticulum; **M**: mitochondria; **(+)** High concentrations; **(−)** Low concentrations. Created with BioRender.com.

*L. pneumophila* exhibits a biphasic life cycle, which is controlled by starvation and environmental stress that induces the transition from metabolically active bacteria to motile, stress-resistant, virulent bacteria [20,39,40]. The biphasic cycle is well described in the host cells' intracellular pathway and resembles the growth curve in an experimental scenario [39,41,42] (Fig. 1). In brief, when conditions are favourable in terms of availability of nutrients and living space, such as within amoebas or macrophages, *L. pneumophila* replicates (exponential growth/replicative state) in a non-motile and non-cytotoxic form. However, when nutrients become limiting, *L. pneumophila* switches to a transmissive phase (post-exponential/stationary growth), resulting in a flagellated, spore-like, and stress-resistant virulent form that can egress the host cells [43], survive planktonically in the environment and re-establish a replicative niche in protozoa and possibly biofilms. Once in the bulk system, *L. pneumophila* exhibits high infectivity, mobility, and cyst-like morphology, and can assume a mature intracellular form (MIF) [44,45] as well as a viable but non-culturable (VBNC) form in response to harsh environmental conditions [46,47] (Fig. 1, Fig. 2).

This complex pleomorphic behaviour is regulated by a set of regulatory systems that control gene expression. Table 1 compiles the main regulatory systems described that play an important role in *L. pneumophila* infection in amoeba and in the biofilm life cycle. Proteomics and transcriptomics studies revealed key metabolic pathways, common to *in vivo* infection models and *in vitro* broth cultures, that dictate the phenotypic shift from the replicative to the transmissive phase [48]. This phenotype transition is coordinated by regulatory systems that control gene expression, such as regulatory proteins (CsrA, RpoS, FliA and FleQ), the LetA/LetS (LetA/S) two-component system (*Legionella* transmission activator and sensor, respectively) the stringent response metabolites (RelA, SpoT and ‘ppGpp’) and noncoding/small RNA (nc/sRNA) [[49], [50], [51], [52], [53]]. During the replicative phase, genes related to metabolism, amino acid degradation, sugar assimilation, cell division and biosynthetic processes are upregulated. In contrast, when bacteria enter a transmissive phase, genes related to virulence, survival, host attachment and ingress are upregulated, including Icm/Dot type IV secretion system (T4SS) (intracellular multiplication/defective for organelle trafficking), motility machinery (flagellar and type IV pilus genes), and cyclic-di-GMP regulatory proteins [42].

**Table 1:** **-** Main molecules that play a crucial role in *Legionella pneumophila* infection in amoeba and in the biofilm.

**Virulencefactors**	**Cellular function**	**Biofilm stage**	**Amoeba impact**	**Biphasic cyclephaseinvolved**^a^	**Reference**
FleQ (sigma factor σ^54^)	The master regulator of the flagellar regulation cascade	Formation	Adhesion; Egress (?)	Transmissive	[52]
FliA (Alternative sigma factor σ^28^)	Important regulatory functions in the flagellar biosynthesis pathway	Formation	Adhesion; Egress (?)	Transmissive	[54,55]
TatB and TaC (Putative twin-arginine translocation pathway)	Transport of folded proteins across the cytoplasmic membrane	Formation	Intracellular replication	Transmissive (?)	[56]
Lcl (*Legionella* collagen-like protein)	Protein that binds to sulfated glucosaminoglycans (CAGs) present in the host extracellular matrix	Formation	Adhesion	Transmissive (?)	[[57], [58], [59]]
LadC	Putative adenylate cyclase is involved in host cell processes	Unknown	Adhesion	Transmissive (?)	[60]
Type IV pili	Inner membrane-associated protein	Formation	Adhesion	Transmissive	[61]
BffA	Involved in the regulation of motility, cellular replication, and virulence	Formation	Unknown	Transmissive (?)	[62]
Type II secretion	Export of various virulence factors involved in bacterial pathogenicity	Formation	Intracellular replication	Nutrient acquisition during replicative phase	[61,64]
Dot/Icm Type 4 Secretion System (T4SS)	Modulates host processes including phagosome-lysosome binding	Unknown	LCV development and intracellular replication	Replicative	[65,66]
EnhC	Immune escape and persistent survival	Unknown	Intracellular replication	Transmissive (?)	[67]
LbtA	Rhizoferrin biosynthetic gene critical for iron acquisition	Formation	Intracellular infection	Replicative	[63]
FeoB	Involved in ferrous iron uptake	Formation	Unknown	Replicative (?)	[63]
PvcAB and AhpC2D	Gene's cluster whose expression is regulated by iron	Protection against oxidative stress	Intracellular replication	Replicative (?)	[68]
SidE effector protein family (SidE, SdeA, SdeB, SdeC)	Modulation of bacterial infection	Unknown	Recruitment of the ER vesicles via ER fragmentation to the LCV; Golgi fragmentation	Transmissive	[68,69]
PmrA/PmrB	Virulence regulator	Unknown	Intracellular replication	Transmissive (?)	[70]
CsrA (Carbon storage regulator A)	Post-transcriptional regulator of gene expression	Affects the expression of the regulators FleQ, RpoS, LqsR	Intracellular replication; pathogenicity	Replicative	[40,50,71]
LetA/LetS (*Legionella* transmission activator and sensor)	Activate expression of two small regulatory RNAs, RsmT and RsmZ	Involved in CsrA expression	Lysosome evasion	Transmissive	[27,72,73]
RsmT/RsmZ (Regulator of Secondary Metabolism)	Relieve the repression of the transmission traits through binding CsrA	Unknown	Intracellular replication	Transmissive	[74,75]
Lqs gene cluster	Regulates the switch from the replicative to the transmissive/virulent phase, pathogen-host cell interactions, cell motility	Could play a role in the dispersion during later stages of biofilm and regulate the phenotypic variation	Intracellular replication; motility	Transmissive (?)	[[76], [77], [78]]
RpoS	Virulence regulator	Regulation of FliA expression	Regulate motility, sodium sensitivity, and evasion of the endocytic pathway	Transmissive	[53,79,80]
Mip	Macrophage infectivity potentiator	Upregulated at the end of biofilm formation	Virulence	Transmissive	[[81], [82], [83], [84]]
LepA/LepB	Non-lytic release	Unknown	Egress	Transmissive (?)	[85]
SpoT and RelA	Synthesis of the alarmone ppGpp	Unknown	Infectivity; motility.	Replicative Transmissive	[86,87]
c-di-GMP	Signalling system involve motility, virulence, the cell cycle, differentiation, and other processes	Biofilm formation and dispersal	Intracellular replication; motility	Transmissive (?)	[88]
ppGpp	Involved in expressing or repressing some regulators, such as FleQ, FliA, RpoS, LqsR, LetA	Unknown	Intracellular replication	Transmissive	[89,90]

a (?) proposed by the authors after the literature review.

In the next section, the molecular mechanisms involved in the regulation of this biphasic life cycle during the intracellular infection cycle in the protozoa will be deeply explored.

## *3* *L. pneumophila* as a successful facultative intracellular pathogen

In the environment, the debate on the *L. pneumophila* survival and morphological states in biofilms is related to the presence of surrounding amoeba host cells, such as *Hartmannella vermiformis* and *Acanthamoeba castellanii*, and this relationship plays a fundamental role in the ecology and pathology of *L. pneumophila* [91]. *L. pneumophila* can also survive outside amoeba, as planktonic cells or within biofilms [54,92,93]. Nevertheless, it is generally accepted that pre-existing biofilms are being used by the bacteria as sheltering niches [93] but *L. pneumophila* requires the protozoa to replicate within water systems [31,33] and this can be the major strategy for their survival in these harsh environments. Because of their near-constant exposure to biofilm-grazing protozoan [91], *L. pneumophila* has been able to adapt and withstand the eukaryotic cells phagocytosis, enabling them to persist and replicate intracellularly [94,95]. When the cytoplasmic environment conditions become less fortunate, the bacteria tend to evade the host cells back into the bulk system [96]. Then, by aerosolization of water particles, the bacteria can be inhaled and trigger pulmonary infections (Fig. 1). The co-evolution with protozoa prompted the adaptation of *L. pneumophila* to human defense cells (macrophages), where it seems to use similar intracellular pathways to persist [21,97]. The macrophages intracellular mechanisms and lung infection are extensively reviewed by Khweek et al. (2010), Newton et al. (2010), Brown et al. (2017) [21,98,99].

### 3.1 The intracellular pathway

Several bacterial factors enhance the initial attachment of *L. pneumophila* cells to the host (Table 1). Notably, the Lcl protein, which is a collagen-like protein that plays a crucial role in protozoa and macrophages adhesion [57], is also reported to be essential in biofilm formation. It facilitates adhesion to abiotic substrates and participates in biofilm cohesion processes of cell-cell/cell-matrix interactions [58,59]. Upon entering the amoeba through phagocytosis mechanisms [[100], [101], [102]], the bacterium avoids lysosome-mediated degradation, loses its flagella, and forms a unique replication-permissive compartment, called the *Legionella*-containing vacuole (LCV). This compartment is surrounded by fragments of host cellular structures such as endosomes, and the endoplasmic reticulum. The LCV allows the bacterium to avoid the host's bactericidal mechanisms and provides a suitable environment for replication (replicative state on Fig. 2 B.2) [21,47]. LCV formation is controlled by the Icm/Dot type IV secretion system (T4SS) (intracellular multiplication/defective for organelle trafficking). This system translocate around 300 effector proteins into host cells [65,66]. These proteins signal transduction, cytoskeletal dynamics, and membrane trafficking, controlling every step of the infection process [65,103]. The initial differentiation from a transmissive stage to a replicative stage is induced by the presence of nutrients, such as amino acids, inside this compartment [104]. When nutrients become limited, bacteria enter the stationary phase and consequently upregulate virulence genes. This transition is a highly coordinated process that is initiated upon nutrient limitation (transition from replicative to transmissive state on Fig. 2 B.3). Amino acid starvation triggers the synthesis and accumulation of guanosine 3,5-bispyrophosphate (ppGpp) [105]. The ppGpp plays a crucial role in recruiting sigma factors allowing the activation of genes required for adapting to the new conditions and repressing those that are no longer necessary. In short, the enzymes RelA and SpoT synthesize ppGpp, resulting in changes in gene expression that lead to phenotypic modulation [87,89,106]. The activation of the alternative sigma factor RpoS, an important virulence factor, results in downstream effects that activate the LetA/LetS two-component system [72]. This system upregulates two small non-coding RNAs, which relieve the repression of virulence-related genes by repressing the global repressor CsrA [40]. Moreover, the sigma factor FliA, an important regulatory function in the flagellar biosynthetic pathway, positively affects the establishment of infection by facilitating the encounter with the host cell, enhancing the invasion capacity, as well as the intracellular replication within *Dictyostelium discoideum* [55,107,108]. Interesting suggestions have been made regarding the role of transcriptional regulators in surface adhesion and biofilm formation, although none of the mutants lacking *rpoS*, *letA*, or *csrA* inhibited biofilm formation in *L. pneumophila* [54]. In fact, only the mutant lacking *flia* showed inhibition of biofilm formation in *L. pneumophila* [54]. After replicative growth (post-exponential phase), when the nutrients of the host cells are exhausted, the bacteria undergo from a metabolically active, non-infectious state to a virulent, transmissive form, completing the biphasic cycle, egressing the amoeba cells and further integrate a new round in the environmental life cycle [20,21,109].

Furthermore, intracellularly, *L. pneumophila* can differentiate into a spore-like, stress-resistant, virulent, and metabolically dormant mature infectious form (MIF) that is released into the cytosol and from the host cell (Fig. 2 B.4) [18,45,110]. Bacterial release can occur via non-lytic and lytic pathways [85]. Chen et al. (2004) showed that the LepA and LepB proteins are involved in the non-lytic release of infected vesicles by protozoa, via an exocytic pathway [85]. However, lysis can be mediated by enzymes with cytolytic or hemolytic activity, such as regiolysin, RtxA, metalloprotease MspA, phospholipase PlaB, or other phospholipases [111]. Moreover, morphological forms can differentiate into the VBNC form and persist in the environment when present in water for prolonged periods [44]. VBNC cells have the potential to resurrect and become culturable under favourable conditions, such as changes in environmental factors or nutrient availability. In addition, VBNC cells can serve as a reservoir for antibiotic resistance genes that can be transferred to other bacteria in the surrounding environment, contributing to the spread of antibiotic resistance in water systems [112].

## 4 Biofilms: a shelter for *L. pneumophila*

The microbial communities in water systems, along with protozoa and other microflora [113], are known to offer protection to *L. pneumophila* [7,19,114] and the bacteria have developed mechanisms to acquire nutrients through the colonization of multispecies biofilms. Instead of attaching directly to the surface as a primary colonizer, it joins pre-existing biofilm [31,117,118]. The crucial role in the ecology of *L. pneumophila* is due to its interaction with the natural microflora [119], which may persist both in the presence and absence of amoeba. Some studies ascertain that *L. pneumophila* can't survive without the presence of amoeba [32,120,121]. A study conducted by Declerck et al. (2009) using a rotating annular reactor to simulate the biofilms in water distribution pipes, found that the presence of *A. castellanii* in the biofilms composed of *Aeromonas hydrophila*, *Escherichia coli*, *Flavobacterium breve* and *Pseudomonas aeruginosa* led to a significantly higher invasion of *L. pneumophila* compared to control experiments where *A. castellanii* was absent [31]. Nevertheless, another study using a rotating disc reactor to grow biofilms on stainless steel coupons, showed that *L. pneumophila* was able to persist in biofilms composed of *P*. *aeruginosa*, *Klebsiella pneumoniae*, and *Flavobacterium* sp., even in the absence of the protozoan host *H*. *vermiformis*. The ability of *L. pneumophila* to replicate was determined by quantifying the loss of plasmid through quantification of the GFP signal. Although, the study does not delve into the detailed processes through which *L. pneumophila* remains in the biofilm matrix without replication when *H. vermiformis* is absent [32]. Additionally, these studies do not explore the potential effect of environmental factors, such as temperature and nutrient availability, on the survival and persistence of *L. pneumophila* in biofilms [31,32,122].

From another standpoint, *L. pneumophila* seems to persist even without the presence of amoeba by using the matrix via acquiring metabolites from other bacteria in the biofilm [19,32,68]. Adding to the discussion, certain bacteria facilitate the enduring persistence and presence of *L. pneumophila* in biofilms, whereas others impede its colonization. For example, *Pseudomonas fluorescens*, *Pseudomonas putida*, *K*. *pneumoniae* and *Flavobacterium* sp., provide a positive effect that is described as an effect of the production of capsular extracellular matrix material by these microorganisms, as well as the availability of nutrients essential for the survival and growth of *L. pneumophila* [93]. On the other side, the presence of other species, such as *P*. *aeruginosa*, *Aeromonas hydrophila*, *Burkholderia cepacia*, *Acidovorax* sp., and *Sphingomonas* sp. [119], play an antagonist role. Stewart et al. (2012) reported another interesting finding that *L. pneumophila* can survive in a three-species biofilm formed by *P. aeruginosa* and *K. pneumoniae* [93]. A previous study suggests that *P. aeruginosa* Quorum Sensing (QS) exerts bacteriostatic and virulence factors by suppressing *L. pneumophila* growth and biofilm formation [123], and it appears that permissive *K. pneumoniae* can overcome this inhibitory effect. This complex survival strategy is controlled by external signalling that triggers specific molecular responses and a complex genetic network.

It is worth noting that multiple studies examining *L. pneumophila* biofilms found in the literature were conducted *in vitro*, some of them using 12-well polystyrene microtiter plates [93,122] and may not accurately reflect real-world aquatic systems. In fact, in some studies, monospecies biofilm of *L. pneumophila* were formed *in vitro*, which has not been proved to exist in natural environments [115,116]. A conceptual scheme is proposed here to outline plausible molecular mechanisms that operate during *L*. *pneumophila* presence in biofilms, including adhesion to pre-established systems, persistence, and dispersal/detachment (see Fig. 2). This could be used to systematize the existing information and provide a starting point for understanding the mechanisms involved in *L. pneumophila* heterogeneity in biofilms.

## 5 Transcriptomic regulation on *L*. *pneumophila* biofilms

It is conceivable that there are similarities between the intracellular and extracellular mechanisms of the bacterium, including the switch between the replicative and transmissive state, and its behaviour when adhering to, persisting in, and releasing from the biofilm. However, there is currently insufficient evidence to support the proposition that morphogenetic changes are similar in both ecosystems. The information compiled on the molecular regulation involved in the *L. pneumophila* biofilms enabled us to establish a framework of the molecular mechanisms involved in the adhesion, maturation, and detachment of *L. pneumophila* biofilms (highlighted in Fig. 2).

It is worth noting that some structured genes involved in protozoa invasion, such as the Lcl protein, Type IV pili, and the FliA, were found to play an important role in biofilm adhesion [54,57,58,61,124]. In addition, it has been found that the global regulator, CsrA, which is involved in downregulation of virulent factors during intracellular infection (transmissive phase), is upregulated in sessile cells [68]. Another noteworthy discovery is the upregulation of *mip* gene expression in the final stage of biofilm as well as in protozoa infection (transmissive phase) [84,125]. These findings suggest that the biofilm provides a favourable environment that protects the replicative state of *L. pneumophila*. However, there are still unanswered questions regarding the regulation of the maturation and dispersion of the *L. pneumophila* biofilm. It is important to deepen our knowledge on these topics.

The upcoming sections will explore the molecular regulation involved in the adhesion of *L. pneumophila* to the biofilm, the different genes present in sessile cells, and the impact of the signalling systems on the architecture and dispersion of the biofilm will be explored.

### 5.1 Molecular mechanisms involved in *L. pneumophila* adhesion to biofilms

Upon attachment to a surface, bacteria undergo morphogenetic changes that initiate the sessile lifestyle and the development of a biofilm matrix, which is contingent upon microenvironmental parameters such as microflora, surface type, and fluid properties [126]. Biofilm formation is a tightly regulated process governed by multiple transcriptomic networks, that are modulated by a wide range of external factors and trigger intricate signalling pathways [25,127]. An essential adhesin protein is the Lcl protein that binds to sulfated glucosaminoglycans (CAGs) present in the host extracellular matrix [58] and also facilitates the adhesion to protozoa and macrophages [57]. It is now well-described as playing a vital role in biofilm formation by facilitating attachment to abiotic substrates and participating in cell-cell/cell-matrix interactions, which is essential to the three-dimensional structure of the biofilm [58,59]. Mallegol et al. (2012), demonstrated differential regulation of Lcl during growth phases and biofilm formation in a static biofilm assay. The mutant lacking Lcl showed impaired adhesion, biofilm formation and intercellular interactions. More interestingly, the down-regulation of Lcl may facilitate the dispersal of *L. pneumophila* to initiate biofilm colonization on another surface [58]. The twin-arginine transport (Tat) secretion is important for the transport of completely folded proteins across the cytoplasmic membrane, and the deletion of *tatB* and *tatC* genes results in a significant reduction in biofilm formation suggesting that this system may facilitate the secretion of specific proteins involved in the early stages of biofilm [56]. Additionally, the *tatB* and *tatC* mutants exhibit impaired intracellular replication and showed a significant impact on intracellular replication in *Acanthamoeba castellanii* [56]. As mentioned in section 3.1 the flagellar sigma factor FliA gene is necessary for the expression of genes associated with the transmissive phase of *L. pneumophila*, including flagella, macrophage infection, and lysosome evasion [124]. Indeed, a mutant lacking *fliA* showed reduced biofilm formation, suggesting a role for FliA in this process [54]. Type IV pili have also been implicated in *L. pneumophila* biofilm colonization based on their role in adherence to protozoan cells [128]. Lucas et al. (2006) showed that type IV pili and the pre-pilin peptidase facilitated *L. pneumophila* colonization of biofilms and the absence of type IV pili resulted in lower attachment levels compared to the wild-type strain. However, the presence of amoeba allowed for attachment and retention of the mutant type IV pili at levels similar to the wild-type strain [61].

### 5.2 Genes involved in *L. pneumophila* biofilms

Hindré et al. (2008), conducted a pioneering biofilm transcriptome analysis of *L. pneumophila,* comparing the replicative and transmissive phases during the growth of *L. pneumophila* in *A. castellanii*. The study demonstrated that biofilm may serve as an apt habitat for *L. pneumophila*, by the expression of genes that repress the transmissive phase in sessile cells [68]. This study showed that the gene encoding the global regulator, CsrA, which is involved in the downregulation of flagellar gene expression and RpoS during a replicative phase [40], was found to be induced in the sessile cells. These findings imply that the regulatory networks are altered in the biofilm in comparison to planktonic life. Additionally, four genes are expressed in the sessile form that comprises two distinct clusters. The PvcAB gene cluster contains the *pvcA* and *pvcB* genes, homologous to the *pvcA* and *pvcB* in *P. aeruginosa* which are necessary for the synthesis of the siderophore pyoverdine. In *L*. *pneumophila*, this cluster is believed to regulate iron metabolism by controlling concentration and facilitating uptake and sequestration below toxic levels [68]. The other group of genes, alkyl hydroperoxide reductases, *ahpD* and *ahpC2*, have been shown to play an influential role in the defence against oxidative stress in the cells of the formed biofilm [68]. Upregulation of *ahpD* and *ahpC2* is documented to be a feature reflecting *L. pneumophila* in its replicative phase [42], and a study performed by Quan et al. (2020), confirms this upregulation after 24 h of the intracellular growth phase of *L. pneumophila* within *Acanthamoeba*. Iron is an essential nutrient for *L. pneumophila* growth and persistence, which tightly controls biofilm formation [117]. Therefore, the metabolism of iron and oxidative stress is related and the induction of these two clusters in sessile cells may help *L. pneumophila* against oxidative stress resulting from high iron concentrations. Another interesting study conducted by Lopez et al. (2023) investigated the role of the rhizoferrin biosynthetic gene (*lbtA*) in *L. pneumophila* biofilms and infection of host cells. The study also explored the possibility of functional redundancy between the rhizoferrin and the ferrous iron transport pathway (FeoB) in iron acquisition. The *lbtA feoB* mutant of *L. pneumophila* was highly defective in forming biofilms on plastic surfaces. This suggests that both the rhizoferrin biosynthesis gene (*lbtA*) and ferrous iron transport gene (*feoB*) are critical for biofilm formation. However, the *lbtA*-containing complement of the mutant was able to restore biofilm formation, indicating that the *lbtA* gene plays a specific role in this phenotype [63]. Besides that, Marin et al. (2022), identified a specific gene, *bffA*, in *L. pneumophila* that appears to be involved in the regulation of motility, biofilm formation, cellular replication, and virulence. The knockout strain lacking *bffA* showed enhanced biofilm formation, reduced motility, enhanced uptake into amoeba, and altered growth kinetics on solid media. This suggests that *bffA* plays a role in signalling pathways that govern changes in growth rate and motility in response to environmental conditions [62]. Furthermore, Andreozzi et al. (2014) showed that the expression levels of the macrophage infectivity potentiator (*mip*) were constant during the early stages and upregulated at the final stage of biofilm formation [84]. These results are similar to the behaviour of *L. pneumophila* in the infection cycle in human protozoa and macrophages. In fact, *mip* gene expression is downregulated during the early stages of infection (replicative phase), but upregulated in the final stages during host cell escape (transmissive phase) [125].

#### 5.2.1 Signalling systems that regulate *L. pneumophila* biofilms

A variety of small signalling molecules generally mediates cell-cell communication [78]. Quorum sensing is the production and release of chemical signal molecules (autoinducers) that control bacterial group behaviour [78,129]. Quorum sensing is the regulation of gene expression of several bacterial processes and behaviour in response to changes in population density, including virulence, sporulation, and biofilm formation/detachment [78,130]. In these communication processes, *L. pneumophila* uses the Lqs (*Legionella* quorum sensing) system, which comprises the autoinducer synthase LqsA, the sensor histidine kinases LqsS and LqsT and the response regulator LqsR [[131], [132], [133]]. At high cell density, the autoinducers accumulate and trigger a coordinated response by binding specific receptors [130]. This system responds to LAI-1 (3-hydroxypentadecane-4-one), a molecule involved in the regulation of virulence, cellular phase switch between the replicative/and the transmissive phase, and motility, among others [134]. LAI-1 is an α-hydroxyketone (AHK) and has been identified in *L. pneumophila* as a quorum-sensing molecule. These molecules have been described as being able to indirectly influence *L. pneumophila* biofilm colonization, production of extracellular filaments and sedimentation through the Lqs system [135]. Moreover, the bacterium's response to environmental changes is linked to the molecule cyclic di-GMP (c-di-GMP), which is a crucial signalling system involved in various bacterial traits, such as cell cycle, motility, virulence, and biofilm formation and dispersal [[136], [137], [138]]. Planktonic and sessile lifestyles are promoted by low and high intracellular c-di-GMP concentrations, respectively. In fact, c-di-GMP is a key regulator in biofilm dispersion, controlling intracellular levels and influencing enzyme production for matrix degradation, facilitating dispersion. Active dispersion involves reducing c-di-GMP, breaking down the biofilm, and releasing cells [138]. In *L.* *pneumophila*, the c-di-GMP is linked to the Lqs system through the pleiotropic transcription factor, LvbR (*Legionella* virulence and biofilm regulator). This pleiotropic transcription factor is negatively regulated by the sensor kinase LqsS, directly controlling the production of proteins involved in c-di-GMP metabolism, as well as the biofilm architecture and pathogen-host cell interactions [139,140]. Hochstrasser et al. (2022) explored the Lqs-LvbR and c-di-GMP networks in the migration of *Acanthamoeba castellanii* through *Legionella* biofilms. The authors conclude that this regulatory network is directly involved in shaping the biofilm architecture, contributing to the formation of either 'patchy' or 'mat-like' structures. The *lvbR*-deficient strain of *L. pneumophila*, which has impaired c-di-GMP regulation, accumulates less sessile biomass, and forms homogeneous mat-like structures, leading to altered biofilm architecture [88].

Personnic et al. (2021) showed that the sessile *L. pneumophila* exhibits phenotypic heterogeneity and was able to form growing and non-growing (“dormant") bacterial populations, controlled by the Lqs system, the transcription factor LvbR and temperature. Interestingly the non-growing sessile cells showed high metabolic activity, expressed virulence genes, and showed tolerance toward antibiotics. These sessile non-growers may lead to a dormant phase, promoting additional long-term survival capacity in the environmental niche and infecting surrounding protozoa [77]. Another interesting study showed that the LvbR acts as a key regulator of biofilm architecture in *L. pneumophila*, influencing the accumulation of sessile biomass and the formation of compact bacterial aggregates within the biofilm. In fact, the *lvbR*-deficient strain of *L. pneumophila* accumulates less sessile biomass and forms homogeneous mat-like structures, while the wild-type strain develops more compact bacterial aggregates [76].

## 6 Technologies for biofilms transcriptomic analyses

Knowledge of the ecology of bacteria and their three-dimensional positioning within a biofilm has changed considerably over the last few decades, in line with technological developments and the emergence of new tools applied to the science of biofilms, both microscopic imaging and molecular tools applied to systems biology. It is now possible to model the entire three-dimensional biofilm structure, and predict the changes that may occur in response to environmental variations [141]. Nonetheless, it is also possible to perform genotypic and phenotypic analyses of biofilm cells, using “omics” (metagenomics, transcriptomics, and metabolomics) [35,141]. As explored by Azeredo, a deep understanding of the structure of the biofilm as a whole, but also at the single-cell and single-molecule level and of its relationship with the surrounding environment, will open up the development of models that allow reproducible studies, but also its control or eradication [35]. For *L.* *pneumophila* the application of these technologies is not prominent, but on the rise. Table 2 outlines the advantages and disadvantages of a few molecular technologies used to study biofilms in *L. pneumophila*.

**Table 2:** **-** Molecular techniques: brief description of the benefits, downsides, and some biofilms studies on *L. pneumophila*.

**Technique**		**Benefits**	**Downsides**	**References on*****L.pneumophila*****biofilms**
**qPCR***Fluorescence detection during PCR reaction*		Quantification of gene expression levels	Disrupting the 3D structure is require, for DNA extraction	
		Real time detection	Susceptible to inhibitory constituents	
		Multiplex experiments	Doesn't distinguish between live or dead cells^a^	[31,196,197]
		Complementary use with Microarray/RNA-Seq results	Lack of correspondence between GU units and CFU's, probably due to VBNC cells	
**CLSM**	**Fluorescent protein (FP)***Plasmidic insertion of a gene coding for a FP, that is expressed when activated*	Real time detection	Cells physiology may be altered	
		No need for 3D Structures destruction	Expression levels can hinder fluorescence signal	
		Genetically encoded	Genetic manipulation of bacteria may affect cellular physiology and survival	[32,58,63,116,176,198]
		2–4 range of colours on multiplex studies	Biofilm growth in real environments cannot be monitored	
		*in vitro* biofilm studies can be followed		
	**FISH***Fluorescently labeled oligonucleotide probes that specifically targets RNA molecules*	Biofilm growth in real environments can be monitored, at well-defined time points	Requires sample fixation to enhance probe accessibility which hinders real-time monitoring^b^	
		Multiplex studies that can go to dozens of targets discriminated, with the use of spectral imaging (CLASI-FISH and seq-FISH)	Probes need to diffuse through biofilm matrix^c^	[31,193,[199], [200], [201]]
		Bacteria are not genetically modified	Fluorescence signal dependent on the number of ribosomes (rRNA), or expression levels of target genes (mRNA)	
		Can detect not only rRNA (population level) but also mRNA sequences (single cell level)		

a PMA-based approaches can allow the discrimination between live and dead cells [159].

b The use of probe delivery techniques, can overcome the need for fixation steps [195].

c Due to their neutrally charged and synthetic nature, the use of DNA mimics, namely peptide nucleic acid (PNA) may enhance probes diffusion, by the increased resistance to proteases and nucleases and more affinity to the target sequence [178].

Quantitative polymerase chain reaction (qPCR) is a widely used technique for studying and quantifying gene expression. It enables real-time monitoring and fast, high-throughput detection and quantification of target DNA sequences in various matrices [142]. qPCR can also supplement other technologies such as microarrays and RNA-seq analysis, making an important contribution to the study of biofilm ecology [38,68,143,144]. This highlights the importance of using complementary techniques to significantly improve the understanding of biofilms functionality. Furthermore, multiplex optimized qPCR assays are viable for detecting various targets in a sample [145,146]. While this technique is still practiced for analyzing waterborne biofilms at the single-molecule and single-cell level [[147], [148], [149], [150], [151], [152]], as Nisar et al. (2022), that study the association of *L. pneumophila* with free-living amoeba in domestic and hospital water systems [152] interpreting the results remains challenging, primarily caused by substances present in water which act as qPCR inhibitors, such as debris, metal ions and humic acids [153,154]. In addition, the number of cells may be overestimated as there is no distinction between DNA/RNA from live and dead cells [155,156]. The implementation of propidium monoazide (PMA), which solely penetrates non-viable cells by damaging their membrane and impeding DNA amplification through DNA cross-linking, has advanced the application of this technique [37,[157], [158], [159], [160]]. While substantial research has been made focusing species detection, the research of mRNA transcripts has also been updated, and there are some attempts to standardize extraction, amplification, and quantification protocols [[161], [162], [163], [164]]. Various extraction kits are available, but to ensure optimal bacterial cell lysis and sample purification, an appropriate optimization of the extraction protocols is necessary, especially due to possible interferences from the biofilm that may still be present [164,165]. Nevertheless, the RNA extraction in biofilms may be the limiting factor when evaluating spatial organization due to the necessity to disrupt the biofilm structure. The extraction and amplification of genomic material fails to provide accurate insights into the morphological condition of the bacteria and the underlying three-dimensional microbial structures [34,166].

**Fig. 3:**
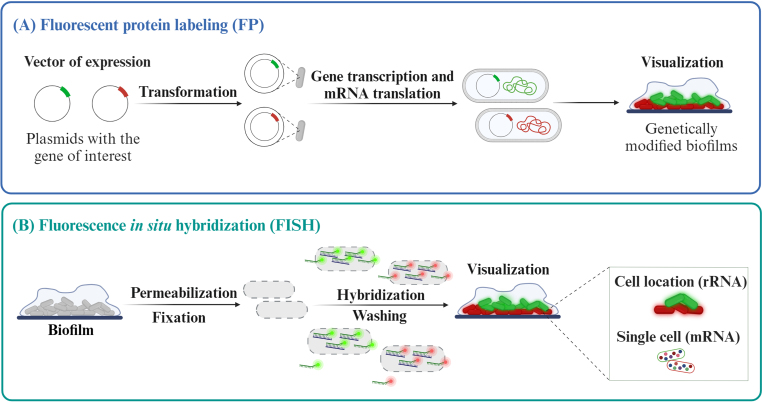
Multispecies biofilm visualization using **(A)** Fluorescent protein labeling (FP) or **(B)** Fluorescence *in situ* hybridization (FISH). Brief description of the protocol for each technique. Created with BioRender.com.

Allied with the technical progress of 3D imaging techniques, which are predominantly based on high resolution microscopy, such as confocal laser scanning microscopy (CLSM), fluorescence-based methods are employed to decode bacterial species patterns within biofilms at a single-cell and molecular levels [166]. These technologies include the expression of fluorescent proteins inserted into bacteria plasmids and fluorescence *in situ* hybridization (FISH) [34]. While both technologies are not superiorly used for *L. pneumophila*, they have already demonstrated their versatility and robustness in biofilm studies (Fig. 3).

The fluorescent proteins (FP) technique is based on the plasmid insertion of a gene encoding an FP, that will be express upon activation [167]. The abovementioned study performed by Murga et al. (2001), applied fluorescent proteins to study the replication capacity of *L. pneumophila* [32]*.* The use of various FPs, such as *GFP* or *mCherry* enables multiplex studies [168,169]. Additionally, this technology, recognized for its high specificity and ability to explore temporal and spatial changes, has been frequently utilized over the past two decades to research gene expression, strain identification, and measure specific biofilm functions in real-time.

The effectiveness of FPs fluorescence may be hindered by its reliance on a minimum O_2_ level during the final stage of chromophore maturation. The distribution of O_2_ in biofilms is uneven since it depends on the balance between gas diffusion through the biofilm and its consumption by the bacteria. This balance determines the spatio-temporal O_2_ distribution, which varies significantly according to the environmental conditions and biofilm stage. To address the issue, a novel O_2_-independent small protein tag called FAST (Fluorescence-Activating and absorption-Shifting Tag) has been used, enabling the reporting of gene expression in oxygen-depleted conditions [170,171]. This technique can widely be applied in water to detect specific bacteria or investigate the physiology of the microbial community [[172], [173], [174], [175]]. In the case of *L. pneumophila*, researchers have drawn parallels between growth within eukaryotic cells and biofilm formation [63,176]. One example of this approach's implementation is laid out in a recent study that investigates the distribution of transmissive *L. pneumophila* on a single-cell level within LCV during the late stages of infection, using FP production [176]. The authors identified a P_*flaA*_-positive transmissive *L. pneumophila* subpopulation, which is controlled by the Lqs system, and becomes visible at the LCV periphery, facilitating the exit and propagation of bacteria outside of the damaged host cell.

FISH is a technique typically based on the complementary targeting of ribosomal RNA (rRNA) by fluorescently labeled oligonucleotide probes [177,178]. For the efficient access of the probe to the target sequence, the cells must be fixed/permeabilized, yet maintaining the cell integrity [178,179]. After the hybridization, the probe will label the target sequence under stringent conditions, exciting the fluorochrome. Generally, it is also performed a washing step, to remove the unbound probe, clearing samples visualization on the microscope [180,181]. Several probes can be used simultaneously, detecting different targets in multiplex approaches [180,182,183]. Nevertheless, these multiplex approaches can be limited to the number of bandpass filters used in imaging equipment's (the same happen to FP technique). However, several FISH variants may be used to increase the number of targets to dozens or hundreds [166]. In a FISH-based technique for combinatorial labeling and spectral imaging (CLASI-FISH), a unique combination of two or more fluorophores, selected to maximize spectral discrimination, are used in a same probe. The targets can be distinguished by the spectral properties of the combined fluorochromes [[184], [185], [186]]. FISH can also be applied to study the spatial localization of cells in mono or multispecies biofilm [180]. Furthermore, it is possible to use FISH-based techniques (e.g. seqFISH, MER-FISH par-seqFISH) to detect single messenger RNA molecules (mRNA), allowing to categorize the metabolic state of the bacteria in different biofilms layers. The spatial transcriptomics methodology is pacing the FISH arena [38,187,188]. A work on spatial transcriptomic analysis on *P. aeruginosa* planktonic and sessile cells was published by Dar and co-workers in 2021 [38]. They developed a parallel sequential FISH (par-seqFISH) to capture cells gene expression profiles, maintaining biofilm structure integrity to study cellular physical and biogeographic context. For *L.* *pneumophila*, gene expression using FISH is a promising area yet to explore, but until the present day, this technique was only applied for *Legionella* detection in water samples [189,190], industrial settings [191,192] and biofilms (species detection) [31,193].

These two techniques are viable in biofilm studies owing to their high signal intensity, ability to distinguish among diverse targets in one experiment, versatility, and specificity. However, each method has distinctive features that imply they are suitable for specific circumstances. FP requires genetic modification of cells, thereby restricting its use in experimental biofilm studies. In FISH, the addition of probes to the biofilm eliminates the need for any prior genetic manipulation, enabling natural biofilm monitoring. Conversely, FP allows for real-time assessment without any invasive cell treatment. On the other hand, FISH entails fixation and permeabilization treatments at each time point, to facilitate probe diffusion through biofilm structure and increase nucleic acid probe target affinity. However, a possible way to overcome this is to combine the use of probe delivery systems [194,195] which are capable of fusing with bacterial envelopes in the absence of fixation chemicals, and synthetic nucleic acid mimics (NAMs), such as Peptide Nucleic Acid (PNA) probes. The shorter length compared to DNA/RNA probes, neutral charge, and chemical structure of the PNA may improve diffusion through the biofilm matrix and enhance target accessibility [178,180,181]. To note that both methods can be combined with other techniques (e.g. qPCR, specific matrix components staining) to provide complementary insights into biofilm spatial organization and bacterial physiology under varying circumstances.

## 7 Conclusions

This review highlights how the understanding of the functionality of a biofilm and the physiology of the cells and their spatial organization is extremely important in order to optimize the means of controlling and eradicating the presence of *L. pneumophila* in biofilms. This is especially significant in the case of this pathogen, as a lack of efficient water system disinfection can lead to severe respiratory infections through bacterial cells aerosolization.

Resembling the biphasic cycle of replicative and transmissive states in amoeba, the bacteria present different morphogenetic states at several phases of biofilm development. These mechanisms are well-documented in the literature, although the possibility of *L. pneumophila* reproducing extracellularly in biofilms without a host still needs a deeper investigation, considering the ecology of the bacterium and the impact of different environmental conditions that the *L. pneumophila* is exposed to in real setting conditions. In this sense, this review provides a holistic description of the different pathways to gain a better understanding of the behaviour of the bacterium as it enters, persists in, or disperses from the biofilm. This study will enhance our comprehension of the bacteria ecological role in aquatic systems. Various methods that can be integrated to explore the genetic expression of the bacterium and its spatial transcriptomics are available, such as qPCR, and 3D imaging techniques allied to fluorescence imaging (FP labeling and FISH).

## Funding

This work was financially supported by: e. Biofilm – “Creation of a group of Excellence on Engineered Biofilms” with the Grant Agreement number 101087568, financed by the 10.13039/501100000780European Commission in the scope of the Horizon Europe Framework Programme; LEPABE, UIDB/00511/2020 (DOI: 10.54499/UIDB/00511/2020) and UIDP/00511/2020 (DOI: 10.54499/UIDP/00511/2020) and ALiCE, LA/P/0045/2020 (DOI: 10.54499/LA/P/0045/2020), funded by national funds through FCT/10.13039/501100006111MCTES (PIDDAC). Ana Barbosa received a PhD fellowship supported by national funds through FCT (grant reference: 2022.11840. BD).

## CRediT authorship contribution statement

**Ana Barbosa:** Writing – original draft, Investigation. **Nuno F. Azevedo:** Writing – review & editing, Supervision, Funding acquisition. **Darla M. Goeres:** Writing – review & editing, Supervision, Funding acquisition. **Laura Cerqueira:** Writing – review & editing, Writing – original draft, Supervision, Resources, Conceptualization.

## Declaration of competing interest

The authors have no competing interests to declare.

## Data Availability

No data was used for the research described in the article.

## References

[1] Mercante J.W., Winchell J.M. (2015). Current and emerging Legionella diagnostics for laboratory and outbreak investigations. Clin Microbiol Rev.

[2] Boamah D.K. (2017). From many hosts, one accidental pathogen: the diverse Protozoan Hosts of Legionella. Front Cell Infect Microbiol.

[3] Mondino S. (2020). Legionnaires' disease: State of the art Knowledge of pathogenesis Mechanisms of Legionella. Annu Rev Pathol.

[4] Bedard E. (2021). Local adaptation of Legionella pneumophila within a hospital hot water system increases tolerance to copper. Appl Environ Microbiol.

[5] van Heijnsbergen E. (2015). Confirmed and potential sources of Legionella reviewed. Environ Sci Technol.

[6] Falkinham J.O. (2015). Epidemiology and ecology of opportunistic premise plumbing pathogens: Legionella pneumophila, Mycobacterium avium, and Pseudomonas aeruginosa. Environ Health Perspect.

[7] Zhang C., Lu J. (2021). Legionella: a promising supplementary Indicator of microbial drinking water Quality in municipal engineered water systems. Front Environ Sci.

[8] Collier S.A. (2021). Estimate of burden and direct healthcare cost of infectious waterborne disease in the United States. Emerg Infect Dis.

[9] Prevention, E.C.f.D. and Control (2019).

[10] Khodr A. (2016). Molecular epidemiology, phylogeny and evolution of Legionella. Infect Genet Evol.

[11] Muder R.R., Victor L.Y. (2002). Infection due to Legionella species other than L. pneumophila. Clin Infect Dis.

[12] Parte A.C. (2020). List of prokaryotic names with standing in nomenclature (LPSN) moves to the DSMZ. Int J Syst Evol Microbiol.

[13] McDade J.E. (1977). Legionnaires' disease: isolation of a bacterium and demonstration of its role in other respiratory disease. N Engl J Med.

[14] Diederen B. (2008). Legionella spp. and *Legionnaires' disease*. J Infect.

[15] Glick T.H. (1978). Pontiac fever. An epidemic of unknown etiology in a health department: I. Clinical and epidemiologic aspects. Am J Epidemiol.

[16] Almeida D.Q. (2021). Outbreak of legionnaires' Disease in the northern Portuguese coast During the COVID-19 pandemic. Acta Med Port.

[17] Hamilton K.A. (2018). Outbreaks of legionnaires' Disease and pontiac fever 2006-2017. Curr Environ Health Rep.

[18] Faulkner G., Garduno R.A. (2002). Ultrastructural analysis of differentiation in Legionella pneumophila. J Bacteriol.

[19] Berjeaud J.M. (2016). Legionella pneumophila: the Paradox of a highly sensitive opportunistic waterborne pathogen Able to Persist in the environment. Front Microbiol.

[20] Oliva G., Sahr T., Buchrieser C. (2018). The life cycle of L. pneumophila: cellular differentiation is linked to virulence and metabolism. Front Cell Infect Microbiol.

[21] Newton H.J. (2010). Molecular pathogenesis of infections caused by Legionella pneumophila. Clin Microbiol Rev.

[22] Yang J.L., Li D., Zhan X.Y. (2022). Concept about the virulence factor of Legionella. Microorganisms.

[23] Gomez-Valero L., Buchrieser C. (2019). Intracellular parasitism, the driving force of evolution of Legionella pneumophila and the genus Legionella. Gene Immun.

[24] Abu Khweek A., Amer A.O. (2018). Factors mediating environmental biofilm formation by Legionella pneumophila. Front Cell Infect Microbiol.

[25] Khweek A.A., Amer A.O. (2019). Biofilm, a cozy Structure for Legionella pneumophila Growth and Persistence in the environment. in Bacterial Biofilms.

[26] Toyofuku M. (2016). Environmental factors that shape biofilm formation. Biosci Biotechnol Biochem.

[27] Chauhan D., Shames S.R. (2021). Pathogenicity and Virulence of Legionella: intracellular replication and host response. Virulence.

[28] Surman S. (2001). Legionella pneumophila proliferation is not dependent on intracellular replication. Legion.

[29] Surman S., Morton L., Keevil C. (1994). The dependence of Legionella pneumophila on other aquatic bacteria for survival on R2A medium. Int Biodeterior Biodegrad.

[30] Wadowsky R.M., Yee R.B. (1983). Satellite growth of Legionella pneumophila with an environmental isolate of Flavobacterium breve. Appl Environ Microbiol.

[31] Declerck P. (2009). Replication of Legionella pneumophila in biofilms of water distribution pipes. Microbiol Res.

[32] Murga R. (2001). Role of biofilms in the survival of Legionella pneumophila in a model potable-water system. Microbiology.

[33] van der Kooij D. (2017). Biofilm composition and threshold concentration for growth of Legionella pneumophila on surfaces exposed to flowing warm tap water without disinfectant. Appl Environ Microbiol.

[34] Costa A.M. (2017). It is all about location: how to pinpoint microorganisms and their functions in multispecies biofilms. Future Microbiol.

[35] Azeredo J. (2017). Critical review on biofilm methods. Crit Rev Microbiol.

[36] Seneviratne C.J. (2020). Multi-omics tools for studying microbial biofilms: current perspectives and future directions. Crit Rev Microbiol.

[37] Magalhães A.P. (2019). RNA-based qPCR as a tool to quantify and to characterize dual-species biofilms. Sci Rep.

[38] Dar D. (2021). Spatial transcriptomics of planktonic and sessile bacterial populations at single-cell resolution. Science.

[39] Molofsky A.B., Swanson M.S. (2004). Differentiate to thrive: lessons from the Legionella pneumophila life cycle. Mol Microbiol.

[40] Molofsky A.B., Swanson M.S. (2003). Legionella pneumophila CsrA is a pivotal repressor of transmission traits and activator of replication. Mol Microbiol.

[41] Faucher S.P., Mueller C.A., Shuman H.A. (2011). Legionella pneumophila transcriptome during intracellular multiplication in human macrophages. Front Microbiol.

[42] Bruggemann H. (2006). Virulence strategies for infecting phagocytes deduced from the in vivo transcriptional program of Legionella pneumophila. Cell Microbiol.

[43] Nora T. (2009). Molecular mimicry: an important virulence strategy employed by Legionella pneumophila to subvert host functions. Future Microbiol.

[44] Robertson P., Abdelhady H., Garduno R.A. (2014). The many forms of a pleomorphic bacterial pathogen-the developmental network of Legionella pneumophila. Front Microbiol.

[45] Garduno R.A. (2002). Intracellular growth of Legionella pneumophila gives rise to a differentiated form dissimilar to stationary-phase forms. Infect Immun.

[46] Hilbi H., Haas A. (2012). Secretive bacterial pathogens and the secretory pathway. Traffic.

[47] Isberg R.R., O'connor T.J., Heidtman M. (2009). The Legionella pneumophila replication vacuole: making a cosy niche inside host cells. Nat Rev Microbiol.

[48] Ge Z. (2022). New global insights on the regulation of the biphasic life cycle and virulence via ClpP-dependent proteolysis in Legionella pneumophila. Mol Cell Proteomics.

[49] Edwards R.L. (2010). The Legionella pneumophila LetA/LetS two-component system exhibits rheostat-like behavior. Infect Immun.

[50] Sahr T. (2017). The Legionella pneumophila genome evolved to accommodate multiple regulatory mechanisms controlled by the CsrA-system. PLoS Genet.

[51] Tiaden A. (2007). The Legionella pneumophila response regulator LqsR promotes host cell interactions as an element of the virulence regulatory network controlled by RpoS and LetA. Cell Microbiol.

[52] Schulz T. (2012). FliA expression analysis and influence of the regulatory proteins RpoN, FleQ and FliA on virulence and in vivo fitness in Legionella pneumophila. Arch Microbiol.

[53] Bachman M.A., Swanson M.S. (2004). Genetic evidence that Legionella pneumophila RpoS modulates expression of the transmission phenotype in both the exponential phase and the stationary phase. Infect Immun.

[54] Mampel J. (2006). Planktonic replication is essential for biofilm formation by Legionella pneumophila in a complex medium under static and dynamic flow conditions. Appl Environ Microbiol.

[55] Heuner K. (2002). Influence of the alternative sigma(28) factor on virulence and flagellum expression of Legionella pneumophila. Infect Immun.

[56] De Buck E. (2005). Legionella pneumophila Philadelphia-1 tatB and tatC affect intracellular replication and biofilm formation. Biochem Biophys Res Commun.

[57] Vandersmissen L. (2010). A Legionella pneumophila collagen-like protein encoded by a gene with a variable number of tandem repeats is involved in the adherence and invasion of host cells. FEMS Microbiol Lett.

[58] Mallegol J. (2012). Essential roles and regulation of the Legionella pneumophila collagen-like adhesin during biofilm formation. PLoS One.

[59] Duncan C. (2011). Lcl of Legionella pneumophila is an immunogenic GAG binding adhesin that promotes interactions with lung epithelial cells and plays a crucial role in biofilm formation. Infect Immun.

[60] Newton H.J. (2008). Significant role for ladC in initiation of Legionella pneumophila infection. Infect Immun.

[61] Lucas C.E., Brown E., Fields B.S. (2006). Type IV pili and type II secretion play a limited role in Legionella pneumophila biofilm colonization and retention. Microbiology (Read).

[62] Marin C., Kumova O.K., Ninio S. (2022). Characterization of a novel regulator of biofilm formation in the pathogen Legionella pneumophila. Biomolecules.

[63] Lopez A.E. (2023). Legionella pneumophila rhizoferrin promotes bacterial biofilm formation and growth within amoebae and macrophages. Infect Immun.

[64] Graham C.I. (2023). Molecular regulation of virulence in Legionella pneumophila. Mol Microbiol.

[65] Lifshitz Z. (2013). Computational modeling and experimental validation of the Legionella and Coxiella virulence-related type-IVB secretion signal. Proc Natl Acad Sci USA.

[66] Berger K.H., Isberg R.R. (1993). Two distinct defects in intracellular growth complemented by a single genetic locus in Legionella pneumophila. Mol Microbiol.

[67] Liu M., Conover G.M., Isberg R.R. (2008). Legionella pneumophila EnhC is required for efficient replication in tumour necrosis factor alpha-stimulated macrophages. Cell Microbiol.

[68] Hindre T. (2008). Transcriptional profiling of Legionella pneumophila biofilm cells and the influence of iron on biofilm formation. Microbiology.

[69] Xie Y. (2023). Mechanism and modulation of SidE family proteins in the pathogenesis of Legionella pneumophila. Pathogens.

[70] Al-Khodor S. (2009). The PmrA/PmrB two-component system of Legionella pneumophila is a global regulator required for intracellular replication within macrophages and protozoa. Infect Immun.

[71] Sahr T. (2012). Deep sequencing defines the transcriptional map of L. pneumophila and identifies growth phase-dependent regulated ncRNAs implicated in virulence. RNA Biol.

[72] Hammer B.K., Tateda E.S., Swanson M.S. (2002). A two-component regulator induces the transmission phenotype of stationary-phase Legionella pneumophila. Mol Microbiol.

[73] Gal-Mor O., Segal G. (2003). The Legionella pneumophila GacA homolog (LetA) is involved in the regulation of icm virulence genes and is required for intracellular multiplication in Acanthamoeba castellanii. Microb Pathog.

[74] Rasis M., Segal G. (2009). The LetA-RsmYZ-CsrA regulatory cascade, together with RpoS and PmrA, post-transcriptionally regulates stationary phase activation of Legionella pneumophila Icm/Dot effectors. Mol Microbiol.

[75] Sahr T. (2009). Two small ncRNAs jointly govern virulence and transmission in Legionella pneumophila. Mol Microbiol.

[76] Hochstrasser R. (2019). The pleiotropic Legionella transcription factor LvbR links the Lqs and c-di-GMP regulatory networks to control biofilm architecture and virulence. Environ Microbiol.

[77] Personnic N., Striednig B., Hilbi H. (2021). Quorum sensing controls persistence, resuscitation, and virulence of Legionella subpopulations in biofilms. ISME J.

[78] Personnic N., Striednig B., Hilbi H. (2018). Legionella quorum sensing and its role in pathogen-host interactions. Curr Opin Microbiol.

[79] Bachman M.A., Swanson M.S. (2001). RpoS co-operates with other factors to induce Legionella pneumophila virulence in the stationary phase. Mol Microbiol.

[80] Dong T., Schellhorn H.E. (2010). Role of RpoS in virulence of pathogens. Infect Immun.

[81] Cianciotto N.P. (1990). A mutation in the mip gene results in an attenuation of Legionella pneumophila virulence. JID (J Infect Dis).

[82] Cianciotto N.P., Stamos J.K., Kamp D.W. (1995). Infectivity of Legionella pneumophila mip mutant for alveolar epithelial cells. Curr Microbiol.

[83] Helbig J.H. (2003). The PPIase active site of Legionella pneumophila Mip protein is involved in the infection of eukaryotic host cells..

[84] Andreozzi E. (2014). Role of biofilm in protection of the replicative form of Legionella pneumophila. Curr Microbiol.

[85] Chen J. (2004). Legionella effectors that promote nonlytic release from protozoa. Science.

[86] Dalebroux Z.D., Edwards R.L., Swanson M.S. (2009). SpoT governs Legionella pneumophila differentiation in host macrophages. Mol Microbiol.

[87] Zusman T., Gal-Mor O., Segal G. (2002). Characterization of a Legionella pneumophila relA insertion mutant and toles of RelA and RpoS in virulence gene expression. J Bacteriol.

[88] Hochstrasser R. (2022). Migration of Acanthamoeba through Legionella biofilms is regulated by the bacterial Lqs-LvbR network, effector proteins and the flagellum. Environ Microbiol.

[89] Dalebroux Z.D. (2010). ppGpp conjures bacterial virulence. Microbiol Mol Biol Rev.

[90] Richards A.M. (2013). Cellular microbiology and molecular ecology of Legionella-amoeba interaction. Virulence.

[91] Shaheen M., Scott C., Ashbolt N.J. (2019). Long-term persistence of infectious Legionella with free-living amoebae in drinking water biofilms. Int J Hyg Environ Health.

[92] Temmerman R. (2006). Necrotrophic growth of Legionella pneumophila. Appl Environ Microbiol.

[93] Stewart C.R., Muthye V., Cianciotto N.P. (2012). Legionella pneumophila persists within biofilms formed by Klebsiella pneumoniae, Flavobacterium sp., and Pseudomonas fluorescens under dynamic flow conditions. PLoS One.

[94] Harb O.S., Gao L.Y., Kwaik Y.A. (2000). From protozoa to mammalian cells: a new paradigm in the life cycle of intracellular bacterial pathogens. Environ Microbiol.

[95] de Felipe K.S. (2005). Evidence for acquisition of Legionella type IV secretion substrates via interdomain horizontal gene transfer. J Bacteriol.

[96] Molmeret M. (2010). Temporal and spatial trigger of post-exponential virulence-associated regulatory cascades by Legionella pneumophila after bacterial escape into the host cell cytosol. Environ Microbiol.

[97] Al-Quadan T., Price C.T., Abu Kwaik Y. (2012). Exploitation of evolutionarily conserved amoeba and mammalian processes by Legionella. Trends Microbiol.

[98] Khweek A.A., Amer A. (2010). Replication of Legionella pneumophila in human cells: why are we susceptible?. Front Microbiol.

[99] Brown A.S. (2017). The regulation of acute immune responses to the bacterial lung pathogen Legionella pneumophila. J Leukoc Biol.

[100] King C.H. (1991). Effects of cytochalasin D and methylamine on intracellular growth of Legionella pneumophila in amoebae and human monocyte-like cells. Infect Immun.

[101] Chang B. (2005). Identification of a novel adhesion molecule involved in the virulence of Legionella pneumophila. Infect Immun.

[102] Prashar A. (2012). Mechanism of invasion of lung epithelial cells by filamentousLegionella pneumophila. Cell Microbiol.

[103] Hubber A., Roy C.R. (2010). Modulation of host cell function by Legionella pneumophila type IV effectors. Annu Rev Cell Dev Biol.

[104] Fonseca M.V., Swanson M.S. (2014). Nutrient salvaging and metabolism by the intracellular pathogen Legionella pneumophila. Front Cell Infect Microbiol.

[105] Hammer B.K., Tateda E.S., Swanson M.S. (2002). A two-component regulator induces the transmission phenotype of stationary-phase Legionella pneumophila. Mol Microbiol.

[106] Dalebroux Z.D. (2010). Distinct roles of ppGpp and DksA in Legionella pneumophila differentiation. Mol Microbiol.

[107] Dietrich C. (2001). Flagellum of Legionella pneumophila positively affects the early phase of infection of eukaryotic host cells. Infect Immun.

[108] Molofsky A.B., Shetron-Rama L.M., Swanson M.S. (2005). Components of the Legionella pneumophila flagellar regulon contribute to multiple virulence traits, including lysosome avoidance and macrophage death. Infect Immun.

[109] O'Connor T.J. (2016). Iron limitation triggers early egress by the intracellular bacterial pathogen Legionella pneumophila. Infect Immun.

[110] Veiga D.F. (2008). Predicting transcriptional regulatory interactions with artificial neural networks applied to E. coli multidrug resistance efflux pumps. BMC Microbiol.

[111] Eisenreich W., Heuner K. (2016). The life stage-specific pathometabolism of Legionella pneumophila. FEBS Lett.

[112] Zhang J. (2018). Impact of biofilm formation and detachment on the transmission of bacterial antibiotic resistance in drinking water distribution systems. Chemosphere.

[113] Muhammad M.H. (2020). Beyond risk: bacterial biofilms and their regulating approaches. Front Microbiol.

[114] Lau H.Y., Ashbolt N.J. (2009). The role of biofilms and protozoa in Legionella pathogenesis: implications for drinking water. J Appl Microbiol.

[115] Pecastaings S. (2010). Sessile Legionella pneumophila is able to grow on surfaces and generate structured monospecies biofilms. Biofouling.

[116] Piao Z. (2006). Temperature-regulated formation of mycelial mat-like biofilms by Legionella pneumophila. Appl Environ Microbiol.

[117] Portier E. (2016). Iron availability modulates the persistence of Legionella pneumophila in complex biofilms. Microb Environ.

[118] Declerck P. (2010). Biofilms: the environmental playground of Legionella pneumophila. Environ Microbiol.

[119] Guerrieri E. (2008). Effect of bacterial interference on biofilm development by Legionella pneumophila. Curr Microbiol.

[120] Donlan R. (2005). Legionella pneumophila associated with the protozoan Hartmannella vermiformis in a model multi-species biofilm has reduced susceptibility to disinfectants. Biofouling.

[121] Lau H., Ashbolt N. (2009). The role of biofilms and protozoa in Legionella pathogenesis: implications for drinking water. J Appl Microbiol.

[122] Bigot R. (2013). Intra-amoeba multiplication induces chemotaxis and biofilm colonization and formation for Legionella. PLoS One.

[123] Kimura S. (2009). Pseudomonas aeruginosa Las quorum sensing autoinducer suppresses growth and biofilm production in Legionella species. Microbiology.

[124] Heuner K., Steinert M. (2003). The flagellum of Legionella pneumophila and its link to the expression of the virulent phenotype. Int J Med Microbiol.

[125] Wieland H. (2005). Intracellular multiplication of Legionella pneumophila depends on host cell amino acid transporter SLC1A5. Mol Microbiol.

[126] Sauer K. (2022). The biofilm life cycle: expanding the conceptual model of biofilm formation. Nat Rev Microbiol.

[127] Abdel-Nour M. (2013). Biofilms: the stronghold of Legionella pneumophila. Int J Mol Sci.

[128] Stone B.J., Kwaik Y.A. (1998). Expression of multiple pili by Legionella pneumophila: identification and characterization of a type IV pilin gene and its role in adherence to mammalian and protozoan cells. Infect Immun.

[129] Miller M.B., Bassler B.L. (2001). Quorum sensing in bacteria. Annu Rev Microbiol.

[130] Rutherford S.T., Bassler B.L. (2012). Bacterial quorum sensing: its role in virulence and possibilities for its control. Cold Spring Harb Perspect Med.

[131] Spirig T. (2008). The Legionella autoinducer synthase LqsA produces an α-hydroxyketone signaling molecule. J Biol Chem.

[132] Tiaden A. (2010). The autoinducer synthase LqsA and putative sensor kinase LqsS regulate phagocyte interactions, extracellular filaments and a genomic island of Legionella pneumophila. Environ Microbiol.

[133] Kessler A. (2013). The L egionella pneumophila orphan sensor kinase LqsT regulates competence and pathogen–host interactions as a component of the LAI-1 circuit. Environ Microbiol.

[134] Schell U. (2016). The alpha-hydroxyketone LAI-1 regulates motility, Lqs-dependent phosphorylation signalling and gene expression of Legionella pneumophila. Mol Microbiol.

[135] Tiaden A., Spirig T., Hilbi H. (2010). Bacterial gene regulation by α-hydroxyketone signaling. Trends Microbiol.

[136] Valentini M., Filloux A. (2019). Multiple roles of c-di-GMP signaling in bacterial pathogenesis. Annu Rev Microbiol.

[137] Hengge R. (2009). Principles of c-di-GMP signalling in bacteria. Nat Rev Microbiol.

[138] Wille J., Coenye T. (2020). Biofilm dispersion: The key to biofilm eradication or opening Pandora's box? Biofilm.

[139] Hochstrasser R., Hilbi H. (2020). Legionella quorum sensing meets cyclic-di-GMP signaling. Curr Opin Microbiol.

[140] Hochstrasser R., Hilbi H. (2019). Migration of Acanthamoeba castellanii through Legionella biofilms. Methods Mol Biol.

[141] Raes J., Bork P. (2008). Molecular eco-systems biology: towards an understanding of community function. Nat Rev Microbiol.

[142] Kralik P., Ricchi M. (2017). A basic guide to real time PCR in microbial diagnostics: definitions, parameters, and everything. Front Microbiol.

[143] Crépin A. (2023). Sensitivity of Legionella pneumophila to phthalates and their substitutes. Sci Rep.

[144] Carlson H.K., Vance R.E., Marletta M.A. (2010). H-NOX regulation of c-di-GMP metabolism and biofilm formation in Legionella pneumophila. Mol Microbiol.

[145] Marín M.J. (2019). Validation of a multiplex qPCR assay for detection and quantification of Aggregatibacter actinomycetemcomitans, Porphyromonas gingivalis and Tannerella forsythia in subgingival plaque samples. A comparison with anaerobic culture. Arch Oral Biol.

[146] Lenz A.P. (2008). Localized gene expression in Pseudomonas aeruginosa biofilms. Appl Environ Microbiol.

[147] Li L. (2015). Transcriptomic changes of Legionella pneumophila in water. BMC Genom.

[148] Liang J., Faucher S.P. (2022). Transcriptomic adaptation of Legionella pneumophila to transient heat shock. Frontiers in Water.

[149] Qin Z. (2007). Role of autolysin-mediated DNA release in biofilm formation of Staphylococcus epidermidis. Microbiology.

[150] Wolf-Baca M., Siedlecka A. (2019). Detection of pathogenic bacteria in hot tap water using the qPCR method: preliminary research. SN Appl Sci.

[151] Koh W. (2013). Multiplication of the waterborne pathogen Cryptosporidium parvum in an aquatic biofilm system. Parasites Vectors.

[152] Nisar M.A. (2022). Molecular screening and characterization of Legionella pneumophila associated free-living amoebae in domestic and hospital water systems. Water Res.

[153] Nappier S.P. (2019). Advancements in mitigating interference in quantitative polymerase chain reaction (qPCR) for microbial water quality monitoring. Sci Total Environ.

[154] Schrader C. (2012). PCR inhibitors–occurrence, properties and removal. J Appl Microbiol.

[155] Donohue M.J. (2021). Quantification of Legionella pneumophila by qPCR and culture in tap water with different concentrations of residual disinfectants and heterotrophic bacteria. Sci Total Environ.

[156] Falzone L. (2020). Droplet digital PCR for the detection and monitoring of Legionella pneumophila. Int J Mol Med.

[157] Krüger N.-J. (2014). “Limits of control”–crucial parameters for a reliable quantification of viable campylobacter by real-time PCR. PLoS One.

[158] Yasunaga A. (2013). Monitoring the prevalence of viable and dead cariogenic bacteria in oral specimens and in vitro biofilms by qPCR combined with propidium monoazide. BMC Microbiol.

[159] Àlvarez G. (2013). Method to quantify live and dead cells in multi-species oral biofilm by real-time PCR with propidium monoazide. Amb Express.

[160] Gensberger E.T., Sessitsch A., Kostić T. (2013). Propidium monoazide–quantitative polymerase chain reaction for viable Escherichia coli and Pseudomonas aeruginosa detection from abundant background microflora. Anal Biochem.

[161] França A., Melo L.D., Cerca N. (2011). Comparison of RNA extraction methods from biofilm samples of Staphylococcus epidermidis. BMC Res Notes.

[162] França A., Cerca N. (2023).

[163] Fleige S. (2006). Comparison of relative mRNA quantification models and the impact of RNA integrity in quantitative real-time RT-PCR. Biotechnol Lett.

[164] França A., Bento J.C., Cerca N. (2012). Variability of RNA quality extracted from biofilms of foodborne pathogens using different kits impacts mRNA quantification by qPCR. Curr Microbiol.

[165] Franca A. (2012). Optimizing a qPCR gene expression quantification assay for S. epidermidis biofilms: a comparison between commercial kits and a customized protocol. PLoS One.

[166] Barbosa A. (2023). Imaging biofilms using fluorescence in situ hybridization: seeing is believing. Front Cell Infect Microbiol.

[167] Snapp E. (2005). Design and use of fluorescent fusion proteins in cell biology. Curr Protoc Cell Biol.

[168] Hansen M.F. (2019). Fluidic resistance control enables high-throughput establishment of mixed-species biofilms. Biotechniques.

[169] Santos S.B. (2020). Bacteriophage-receptor binding proteins for multiplex detection of Staphylococcus and Enterococcus in blood. Biotechnol Bioeng.

[170] Monmeyran A. (2021). Four species of bacteria deterministically assemble to form a stable biofilm in a millifluidic channel. npj Biofilms and Microbiomes.

[171] Monmeyran A. (2018). The inducible chemical-genetic fluorescent marker FAST outperforms classical fluorescent proteins in the quantitative reporting of bacterial biofilm dynamics. Sci Rep.

[172] Schaefer L.M., Brözel V.S., Venter S.N. (2013). Fate of Salmonella Typhimurium in laboratory-scale drinking water biofilms. J Water Health.

[173] Waegenaar F. (2023). Unwanted coliforms can hide in mature drinking water biofilms, grown in full-scale distribution networks. bioRxiv.

[174] Puri D., Fang X., Allison K.R. (2023). Fluorescence-based protocol for revealing cellular arrangement in biofilms. STAR protocols.

[175] Li J., McLellan S., Ogawa S. (2006). Accumulation and fate of green fluorescent labeled Escherichia coli in laboratory-scale drinking water biofilters. Water Res.

[176] Striednig B. (2021). Quorum sensing governs a transmissive Legionella subpopulation at the pathogen vacuole periphery. EMBO Rep.

[177] Amann R.I. (1990). Combination of 16S rRNA-targeted oligonucleotide probes with flow cytometry for analyzing mixed microbial populations. Appl Environ Microbiol.

[178] Nacher-Vazquez M. (2022). The role of Nucleic Acid Mimics (NAMs) on FISH-based techniques and applications for microbial detection. Microbiol Res.

[179] Cerqueira L. (2008). DNA mimics for the rapid identification of microorganisms by fluorescence in situ hybridization (FISH). Int J Mol Sci.

[180] Almeida C. (2011). Discriminating multi-species populations in biofilms with peptide nucleic acid fluorescence in situ hybridization (PNA FISH). PLoS One.

[181] Cerqueira L. (2013). Biofilm formation with mixed cultures of Pseudomonas aeruginosa/Escherichia coli on silicone using artificial urine to mimic urinary catheters. Biofouling.

[182] Cerqueira L. (2013). Validation of a fluorescence in situ hybridization method using peptide nucleic acid probes for detection of Helicobacter pylori clarithromycin resistance in gastric biopsy specimens. J Clin Microbiol.

[183] Thurnheer T., Gmür R., Guggenheim B. (2004). Multiplex FISH analysis of a six-species bacterial biofilm. J Microbiol Methods.

[184] Azevedo A.S. (2022). Spectral imaging and nucleic acid mimics fluorescence in situ hybridization (SI-NAM-FISH) for multiplex detection of clinical pathogens. Front Microbiol.

[185] Valm A.M., Welch J.L.M., Borisy G.G. (2012). CLASI-FISH: principles of combinatorial labeling and spectral imaging. Syst Appl Microbiol.

[186] Valm A.M. (2011). Systems-level analysis of microbial community organization through combinatorial labeling and spectral imaging. Proc Natl Acad Sci USA.

[187] Chen K.H. (2015). Spatially resolved, highly multiplexed RNA profiling in single cells. Science.

[188] Lubeck E. (2014). Single-cell in situ RNA profiling by sequential hybridization. Nat Methods.

[189] Moreno Y., Moreno-Mesonero L., García-Hernández J. (2019). DVC-FISH to identify potentially pathogenic Legionella inside free-living amoebae from water sources. Environ Res.

[190] Nácher-Vázquez M. (2022). Development of a novel peptide nucleic acid probe for the detection of Legionella spp. in water samples. Microorganisms.

[191] Kirschner A.K. (2012). Development of a new CARD-FISH protocol for quantification of Legionella pneumophila and its application in two hospital cooling towers. J Appl Microbiol.

[192] Zeybek Z., Gungor N.D., Turetgen I. (2017). Investigation of heterotrophic bacteria, legionella and free-living amoeba in cooling tower samples by fish and culture methods. European Journal of Biology.

[193] Wilks S.A., Keevil C.W. (2006). Targeting species-specific low-affinity 16S rRNA binding sites by using peptide nucleic acids for detection of Legionellae in biofilms. Appl Environ Microbiol.

[194] Zhang Y. (2020). Multi-targeted antisense oligonucleotide delivery by a framework nucleic acid for inhibiting biofilm formation and virulence. Nano-Micro Lett.

[195] Moreira L. (2022). Liposome delivery of nucleic acids in bacteria: toward in vivo labeling of human microbiota. ACS Infect Dis.

[196] Taylor M.J., Bentham R.H., Ross K.E. (2014). Limitations of using propidium monoazide with qPCR to discriminate between live and dead Legionella in biofilm samples. Microbiol Insights.

[197] Buse H.Y. (2017). Effect of temperature and colonization of Legionella pneumophila and Vermamoeba vermiformis on bacterial community composition of copper drinking water biofilms. Microb Biotechnol.

[198] Buchrieser C., Hilbi H. (2019). Legion: Methods and Protocols.

[199] Declerck P., Ollevier F. (2006). Detection of Legionella in various sample types using whole-cell fluorescent in situ hybridization. Diagnostic Bacteriology Protocols.

[200] Declerck P. (2007). Replication of Legionella pneumophila in floating biofilms. Curr Microbiol.

[201] Gião M.S. (2011). Interaction of Legionella pneumophila and Helicobacter pylori with bacterial species isolated from drinking water biofilms. BMC Microbiol.

